# A Novel Sensing Method and Sensing Algorithm Development for a Ubiquitous Network

**DOI:** 10.3390/s100908129

**Published:** 2010-08-30

**Authors:** Hamid Jabbar, Sungju Lee, Seunghwan Choi, Seunghyun Baek, Sungwook Yu, Taikyeong Jeong

**Affiliations:** 1 Department of Electronic Engineering, Myongji University, Seoul, Korea; E-Mails: hamid@mju.ac.kr (H.J.); shbeak12@mju.ac.kr (S.B.); 2 Department of Computer & Information Science, Korea University, Seoul, Korea; E-Mail: peacfeel@korea.ac.kr; 3 Department of Software Engineering, Korea Electric Power Corporation, Korea; E-Mail: kepchoi@kepri.re.kr; 4 Department of Electronic Engineering, Chungang University, Seoul, Korea; E-Mail: sungwook@cau.ac.kr

**Keywords:** sensing circuits, ubiquitous network, wireless sensor network, low-power, sensing algorithm

## Abstract

This paper proposes a novel technique which provides energy efficient circuit design for sensors networks. The overall system presented requires a minimum number of independently communicating sensors and sub-circuits which enable it to reduce the power consumption by setting unused sensors to idle. This technique reduces hardware requirements, time and interconnection problems with a supervisory control. Our proposed algorithm, which hands over the controls to two software mangers for the sensing and moving subsystems can greatly improve the overall system performance. Based on the experimental results, we observed that our system, which is using sensing and moving managers, the four sensors required only 3.4 mW power consumption when a robot arm is moved a total distance of 17 cm. This system is designed for robot applications but could be implemented to many other human environments such as “ubiquitous cities”, “smart homes”, *etc.*

## 1. Introduction

All of the sophisticated and intelligent man made equipment need some sort of sensor to infer the environment. Collecting these data from different sensors and to perform the required tasks in a minimum time requires the efficient use of minimum size and easy to install and use sensors, communications and power systems. The above features should be implemented in such a way that there is no loss of required data and redundancy other than the safety.

This paper presents a sensing technique using an experimental platform for micro-sensors used in robot arm for various missions such as space and home networks. Proximity sensors are used in these sorts of applications to determine objects, locations of pins, holes and edges in equipment with sufficient accuracy to permit alignment prior to docking and berthing. The use of several sensors in an array with multiple connections complicates the construction and movement, especially when changing the sensors.

This paper presents a technique to efficiently use four given sensors at the front of a robot arm to detect the object and perform the required task like forcing a tool. The proximity type sensor used in this system has proven its reliability in past space missions [1]. The presented algorithm uses software- based management routines, in which managers govern the multiple sensors and actuators to make an energy efficient system by allowing a minimum number of sensors to be working at any given time. These managers use bidirectional wireless communication among the sensors on the robot arm and the management system to detect the objects and move the robot in a minimum time. The system was tested using a Mote type experimental platform with computational and communication capabilities. By organizing the system power structure and interconnections for stable power and other actuator driving mechanisms with fast response and distributed power we can achieve greatly improved overall system performance.

One issue in smart sensor networks is achieving efficient operation because of the limited available power. A smart sensor is a collection of integrated sensors and electronics. When these types of sensors are used in Wireless Sensor Networks (WSNs), very powerful, versatile networks can be created and used in situations where traditional wired networks fail [2]. In our proposed system using the 2-D network with -four neighbors consumes less power than 2-D networks with six and eight neighbors. This is because of the tradeoffs between the number of neighbors and the power dissipated in the system.

Methodology of the system is described in Section 2. The proposed algorithm and overall system approach is in Section 3. Section 4 describes the experimental platform used in the testing of algorithm and technique, and reports experimental results. Section 5 describes related work, and Section 6 summarizes the whole theme.

## 2. Methodology

### 2.1. Overall Method

This section expresses the sensing methodology of the sensor system; sensors are used to precisely locate an object or pattern of an object using its edges and known locations. For our design we assume frequency information from each of four sensors is from one point of object and each sensor value varies with the distance from the object. The nearer the sensor to the object, the smaller is this frequency value.

**Figure 1.**
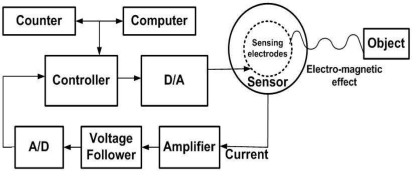
An overall structure of sensing system.

A multiple-element space robot low power sensor with sensing electrodes is used, which is programmed with a proposed sensing algorithm. The capacity type proximity sensor used, comprises a capacitance type sensor, a capacitance type reference, and two independent and mutually opposing driven shields, respectively, adjacent to the sensor and reference and which are coupled in a bridge circuit configuration and driven by a single frequency controlled oscillator [3]. Two mutually opposing driven shields and electro-magnetic effects between the sensing circuit and the sensed object gives rise to a current in the sensors, as shown in Figure 1. Some of the built-in low power sensors, the controller that drives the sensing electrodes, put out a voltage that varies with this current by virtue of the voltage drop of this current across the voltage follower resistor. This voltage follower output is converted to the digital value for control purposes and fed to the controller. The controller of each sensor is capable of transmitting this data to the computer and to the counter so that it can be used by the overall system to guide the robot arm. The controller controls the sensor using feedback through a digital to analog converter. Our controller of the system consists of the Mote circuit board with processor and Tiny OS (operating System) [4]. Section 4 describes in detail the experimental platform used in the testing.

### 2.2. Sensors Detection Methodology

**Figure 2.**
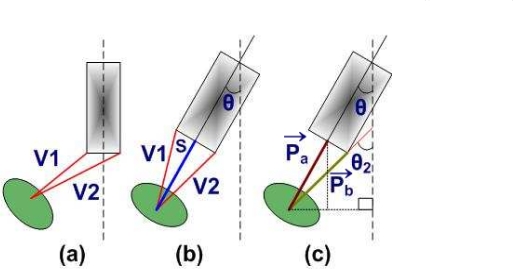
Sensors detection process in 2-D with *P⃗_a_* and *P⃗_b_*.

First we think only in 2-D configuration. *v1* is sensor1 and *v2* is sensor2 known frequency values and current coordinate of sensors are known from hardware feedback. To find an object we increase *v1* and decrease *v2* till we get the angle θ (direction) and S (required frequency), as shown in Figure 2(b). Figure 2(c) shows that S and is *P⃗_a_* (pattern) and angle θ2 for sensor *v2* is *P⃗_b_* (pattern).

**Figure 3.**
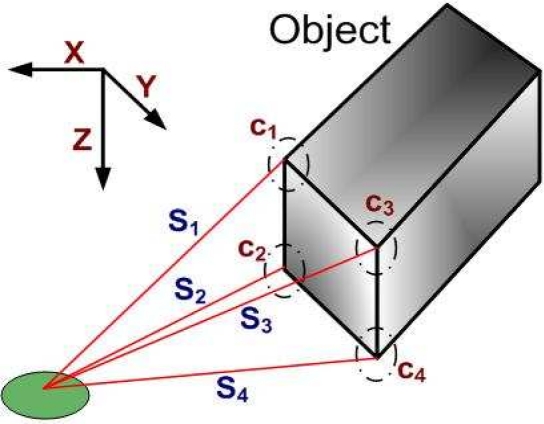
Sensor and object detection in 3-D.

Now we visualize the system in 3-D (as shown in Figure 3). We assume, *c*_1_, *c*_2_, *c*_3_, *c*_4_, and each has the coordinates (*x*_1_, *y*_1_, *z*_1_), (*x*_2_, *y*_2_, *z*_2_), (*x*_3_, *y*_3_, *z*_3_), (*x*_4_, *y*_4_, *z*_4_), respectively. Each sensor has the scalar value *S*_1_ of *c*_1_, *S*_2_ of *c*_2_, *S*_3_ of *c*_3_ and *c*_4_ of *S*_4_. We get the following sets of the equations:

(1) $${\left(x1-x0\right)}^{2}+{\left(y1-y0\right)}^{2}+{\left(z1-z0\right)}^{2}-S_{1}^{2}=0$$

(2) $${\left(x2-x0\right)}^{2}+{\left(y2-y0\right)}^{2}+{\left(z2-z0\right)}^{2}-S_{2}^{2}=0$$

(3) $${\left(x3-x0\right)}^{2}+{\left(y3-y0\right)}^{2}+{\left(z3-z0\right)}^{2}-S_{3}^{2}=0$$

(4) $${\left(x4-x0\right)}^{2}+{\left(y4-y0\right)}^{2}+{\left(z4-z0\right)}^{2}-S_{4}^{2}=0$$

where *x*0, *y*0, *and z*0 ≥ 0.

Then, we can find object coordinate using robot arm origin (*x*_0_, *y*_0_, *z*_0_ ) from the above four equations. Now as we know object locations, we can find pattern *P⃗_a_* using Equations (5) and (6):

(5) $$\vec{U_{p_{a}}}=\sqrt{\left((\frac{\left(x1+x2+x3+x4\right)}{4}-x0),(\frac{\left(y1+y2+y3+y4\right)}{4}-y0),(\frac{\left(z1+z2+z3+z4\right)}{4}-z0)\right)}=0$$

(6) $$S_{p_{a}}=\sqrt{\left({\left(\frac{\left(x1+x2+x3+x4\right)}{4}-x0\right)}^{2},{\left(\frac{\left(y1+y2+y3+y4\right)}{4}-y0\right)}^{2},{\left(\frac{\left(z1+z2+z3+z4\right)}{4}-z0\right)}^{2}\right)}=0$$

(7) $$\vec{P_{a}}=\vec{U_{p_{a}}}\cdot S_{p_{a}}=0$$

Based on the pattern *P⃗_a_*, and we can now guide the robot arm in the direction of the object for an accurate positioning. Using one more sensor to detect the object for proper alignment, we can perform the required task by making some rotation and forcing along the *z*-axis. Also, to clearly explain our methodology, we made a list of 2-D and 3-D configurations as described in Table 1.

## 3. Overall System and Proposed Algorithm

### 3.1. Overall System Design

The combination of a sensing algorithm with feature recognition requires pre-programmed prior knowledge and operator supervision [5]. The micro energy device’s sensing system consumes low power over the whole electrical system and achieves self- recovery via a sensing manager and a moving manager. When we look at sensors for robots performing precision assembly, an operating principle of the micro energy systems relies on a stable power distribution [6]. We also confirmed that contact surface proximity images can be generated at every sensing step because: (i) the end-unit of the robot is centered and aligned with respect to the fastener; (ii) the electromagnetic effect between the end-unit of robot and fastener can be measured; (iii) the range between the tool and fastener can be calculated from simulation and software models, given the capacitance between them; (iv) each sensors’ surface image is derived from the measurement-adjusted simulation model and displayed in oscillation circuit modeling.

**Figure 4.**
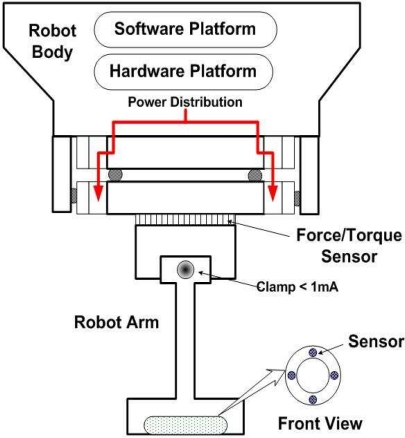
How each sensing mode can apply to the end-unit by sensing manager and moving manager.

As demonstrated by the profiling result of the combination of multiple sensors (shown in Figure 4), our objective is to minimize the sensing power distribution and moving time. This amounts to maximizing the amount of power distribution that flows a current. Since all array sensors are enclosed by a robot body, they contribute the same to the overall power consumption rate as well as moving time using two software managers—the sensing manager and the moving manager. The specific design application can be implemented for sensor network applications and it will be investigated using an electronic design automation tool, including timing constraints and clock/data frequency recovery circuits under the worst-case scenario. The overall goal of the energy efficient sensor circuit is to model the studied system or plant in robots, to design the control system, and to simulate the entire system by embedding the sensor circuits into the robots. The interface to circuits available in the robots system makes it possible to test the control algorithm with detailed models of all of the hardware components, including piezoelectric sensor, actuators, and devices.

A proposed sensor design gathers information from the environment through measuring mechanical, thermal, chemical, biological, optical, and magnetic phenomena. Moreover, sensor applications and fast data processing are in increasing demand. Energy efficient sensor circuit design also allows for the realistic deployment of highly articulated robots into unknown environments and into environments that are too difficult to model. While carrying on sensors, an issue is extremely important the robot’s ability to determine its location in the world.

### 3.2. Proposed Sensing Algorithm

**Figure 5.**
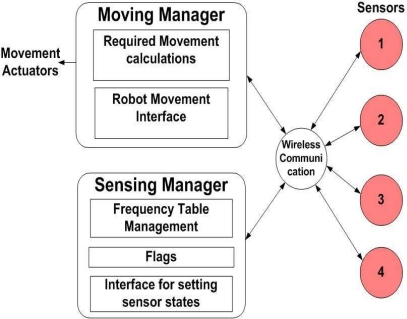
Description of the sensor detection system with moving and sensing manager.

Figure 5 shows how four sensors can detect objects and communicate not only with each other, but also with the managers in our proposed algorithms. We propose a “sensing manager” for controlling the sensors and managing information of each sensor, and a “moving manager” for the actual robot movement.

The four sensors are only needed to read the frequency for a desired object. They send the frequency information to the sensing manger and receive the WAKE or SLEEP commands. Sensors communicate with each other and managers using wireless communication.

The sensing manger has information tables to find the direction and distance of the location. The sensing manager can manage the following tasks and tables:

- Each sensors’ maximum frequency table.
- Each sensors’ minimum frequency table.
- Each sensors’ detection table constructed by Δx, Δy, Δz between sensor and object.
- Each sensors’ state table constructed by the current location x, y, z.
- Each sensors’ flag.
- Each sensors’ state (SLEEP or WAKE).

The moving manger decides and implements the direction, rotation and movement of the robot. It also performs the tasks below:

- Decide first direction pattern (*P⃗_a_*) using four sensors
- Decide similarity direction pattern (*P⃗_b_* ) using one sensor
- Decide angle of desired object using two sensors
- Moving the end-effector
- Stopping the end-effector

Our algorithm, which resides in the Tiny OS of our controller, describes how managers work with four sensors in five different modes initial, moving, self-locking, level-setting and forcing. We discuss each mode and its algorithm in detail.

- Initialization Mode: This mode sets the start point for the robot and tries to find the first direction or pattern (*P⃗_a_*) of the object by awaking all sensors. This Initialization mode algorithm is as follows:

**Table:** 

**Algorithm #1**
**x, y, z = distance (or frequency)**
*P⃗_a_***= pattern (the vector between four sensors and object)**
*P⃗_b_***= Near one sensor to the object**
**init sensing mode{**
**Set the start point x0, y0, z0**
**Find the Δx, Δy, Δz**
*P⃗_b_***= Find Pattern(x, y, z)**
**}**
- Moving Mode: This mode is for finding the similar pattern of the object with other sensors (*P⃗_b_*). After finding the desired object, we need to decide the most energy-efficient, shortest and minimum time-consuming direction before the actual moving mode. For this, we compare the two patterns *P⃗_a_* and *P⃗_b_*. The “sensing manager” has tables of information for each sensor, and updates each table by event. Above steps are repeated until the sensor can find the edge of the object within the shortest distance and robot is stopped. Algorithm #2 shows this moving mode.

**Table:** 

**Algorithm #2**
**while (***P⃗_a_*≠*P⃗_b_***)***P⃗_b_***= Find pattern(x, y, z){**
**put other sensors to SLEEP**
**if( table sensor frequency < current sensor frequency)**
**update “sensing manager” by current sensor frequency**
**}**
**while (table sensor frequency < current sensor**
**frequency){**
**“mobbing manager” decides x, y, z**
**“mobbing manager” moves the robots**
**}**
- Self-locking Mode: All sensors are awake again and we start moving the robot by making all sensors detect the edge of the object at the shortest distance. The above steps are repeated until each sensor’s maximum frequency converges. If it converges, the moving manager stops moving the robot right above the object. Self locking mode is described by Algorithm #3.

**Table:** 

**Algorithm #3**
**while (current sensor frequency = max sensor frequency) {**
**“sensing manager” wakes four sensors**
**“mobbing manager” decides x, y, z**
**“mobbing manager” moves the robots**
**If(table sensor frequency < current sensor frequency)**
**Update “sensing manager” by current sensor frequency**
**Update “flag” by TRUE**
**If(table sensor frequency >= current sensor frequency)**
**Update “flag” by TRUE**
**If(flag = TRUE)**
**“moving manager” increases x, y, z**
**“moving manager” moves the robots**
**If(flag = FALSE)**
**“moving manager” decides moving amount in different directions**
**If(current sensor frequency = max of sensor frequency)**
**Stop moving the robot**
**}**
- Level-setting Mode: the sensing manager decides which two sensors have maximum frequency and minimum frequency values. Except for these two sensors, the sensing manager makes the other two sensors go to sleep. The sensing manager decides the angle with the two awake sensors for level setting and sends the value of the angle to the moving manager, and then the moving manager can do level setting until the robot and object are parallel. Algorithm #4 shows this level-setting mode.

**Table:** 

**Algorithm #4**
**Sensing manager decides two sensors**
**one with max of frequency or SF**
**second with min of frequency or SF**
**current angle = Find angle(maxSF,**
**minSF)**
- Forcing Mode: the sensing manager chooses one sensor and makes another sensor go to sleep. The moving manager moves the robot along the z axis until the robot cannot move any more in this direction. Finally, the moving manager rotates the robot in the z-axis until the sensor can find the matching pattern of the object at the minimum distance. When the sensor finds the pattern, the robot stops rotating and forces the object. As shown Algorithm #5, we described the forcing mode.

**Table:** 

**Algorithm #5**
**Chooses one sensor{**
**Put other sensor to SLEEP**
**Find the matching pattern of object in the minimum distance**
**Forcing the z axis**
**}**

## 4. Results and Discussion

In this section, we confirm the performance of the proposed algorithms and the techniques. For that, we first establish the environment of the platform, and then measure some performance features such as power consumption and execution time.

### 4.1. Experimental Platform

All the proposed algorithms and techniques were tested using an experimental platform consisting of four mote type boards each mounted with a sensor. The Mica2 mote, developed at UC Berkeley [7] is one of the most popular sensor node designs. It is based on a 7.3 MHz Atmel ATmega128 L embedded controller with 4 Kbytes of RAM and 128 K bytes of ROM. The Mica2 includes a low-power, single-chip radio (the ChipconCC1000) capable of operating at 76.8 kbps with a practical indoor range of approximately 20–30 m. The Mica2 dimensions are 5.7 cm, 3.2 cm, 2.2 cm, and uses two AA batteries that will last for up to a week if the device is powered continuously. However, you can extend its lifetime to months or years through careful duty cycling. The Mica2 runs a specialized operating system, called TinyOS, which addresses the sensor nodes concurrency and resource management [8].

**Figure 6.**
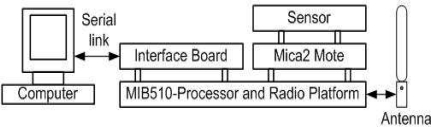
Illustration of Mote Circuit Boards and connections.

Each board has three parts, as shown in Figure 6, which includes a programming board—MIB510 was used for programming the motes and for housing the base mote for wireless communication. Also, Motes-MICA2 includes a processor that runs the Tiny-OS. It is capable of radio transmission and reception. It has a 51-pin connector for housing the sensor. Finally, Sensors-MTS300 has the capability to sense data and transmits it using the processor/radio module. We have programmed the board using nesC language. Board communicate with the UNIX based PC using serial communication.

### 4.2. Simulation Results

For analysis and comparison we analyzed three types of output data from the simulation result we performed. One is the figure of merit, Power Delay (PD) Product, which is essentially the energy consumption per cycle and will remain a constant for a specific machine. Second one is Cache Hit Rate, average time to access memory and tells effectiveness of the cache. Third is the Moving time, the time taken by mode to move the robot or actuator.

We have tested the sensor algorithm using the experimental platform and verified it by benchmarking, using Integer Matrix Multiply (IMM) and Vector Matrix Multiplier (VMM) benchmarks on a machine equipped with an Intel Itanium microprocessor. IMM was chosen because it is designed to facilitate comparing, validating, and improving reconfigurable computing systems. It also provides an architecture-independent compilation framework to express each algorithm’s dependencies and to support automatic synthesis, partitioning, and mapping to a reconfigurable computer [9]. VMM analog VLSI implementation function offers the potential of significant benefits in terms of power, volume, and performance compared to a digital implementation for applications involving fixed or slowly varying weights [10]. Also, in digital systems, VMM reduces the truncation errors and improves vector to vector multiplication.

The benchmark results of our approach on efficient energy consumption are shown in Table 2. We observe our approach for distance moved, active time, power consumption and PD Product in all modes. We note that, since moving mode has more PD Product than others, overall our proposed approach depends mainly on the moving mode, so the proposed algorithm will not restrict the attached hardware actuators’ movements.

**Figure 7.**
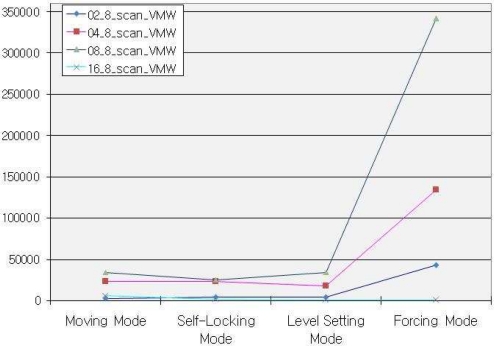
Hardware utilization in each of the different four modes.

**Figure 8.**
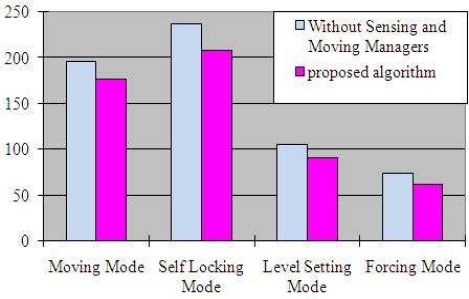
Comparison Results of the Proposed Algorithm and without Sensing and Moving Managers.

Furthermore, for designing of sensors that have more efficient performance in our approach, we observe the logic gates in Figure 7 used by hardware in each mode by keeping data width 8 and varying the parallel processing nodes and hardware cycles. Figure 8 compares the total time taken in each mode of proposed algorithm with the algorithm lacking the Sensing and Moving manager features.

By studying the two data values of Cache Hit Rate and PD Product, we can predict that our algorithm is energy efficient and has low power consumption. Also, from the values of time taken by each mode we are confident that proposed algorithm has no timing constraints and can also perform well in embedded environment with low speed processors.

**Figure 9.**
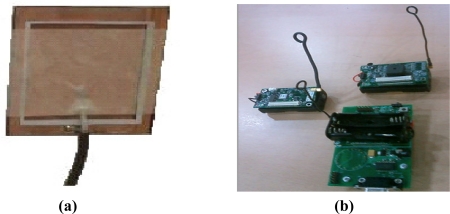
**(a)** A board that installed a micro senor circuit, **(b)** A real experimental sensor device, MOTES-MICA2, including sensing manger and moving manager.

Finally, Figure 9 shows our overall implemented system. Figure 9(a) is a board that included a micro-sensor circuit and Figure 9(b) is a real experimental sensor device, MOTES-MICA2, containing a sensing manger and moving manager. Each sensing and moving manager system was implemented by the embedded board and the robot arm has four sensors. Then the sensing and moving manager system communicates with frequency to the four sensors, and acquires the moving distance, required power consumption, and execution cycle. To precisely adapt the proposed system as well as sensing algorithm, we confirmed that sensor circuits are made by private companies and other defense related industries, e.g., Department of Defense and Aerospace companies. Note that this proposed system could not only be applied to a robot arm, but could also be implemented to many other human environments such as “ubiquitous cities”, “smart homes”, and Wireless Sensor Networks (WSNs), *etc.*

## 5. Related Works

The application of technique presented here is not only limited to robots, as this system can be utilized in a vast range of ubiquitous sensor and network uses. The most promising areas are *ad-hoc* sensor networks for emergency medical care, ubiquitous cities or U-cities and smart homes, on road vehicle detection, *etc*. One example from the use of a sensing device and operating system is early detection of information by Tiny OS [11], for example, in detecting a fire or an alarm warning for a disaster, *etc.*

The system presented uses only one type of sensor, performing the same task. To generalize this technique for future prospects we need to take in account the different type of sensors transmitting different environmental data. We also need to develop a technique to use multiple communication protocols as system can interact with different sensors or equipment which have different communication methods.

There are many sensor manufacturers and many networks on the market today. It is too costly for manufacturers to make special transducers for every network on the market. Different components made by different manufacturers should be compatible. Therefore, in 1993 the IEEE and the National Institute of Standards and Technology (NIST) began work on a standard for Smart Sensor Networks. IEEE 1451, the Standard for Smart Sensor Networks was the result [12]. The objective of this standard is to make it easier for different manufacturers to develop smart sensors and to interface those devices to networks.

## 6. Conclusions

Improving performance is of great importance in modern semiconductor design technology as well as in the system integration area. In this paper, an energy efficient micro-sensor design for robots with an adaptive sensor design method and a new algorithm is proposed, which utilizes the sensor circuit more efficiently to achieve minimum moving time and power consumption. In particular, our paper proposes a method to determine the performance and sensing algorithm by integrated sensor and low-power circuits. Based on the experimental result, we observed that with our system, using a sensing and a moving manager, the four sensors required only 3.4 mW power consumption when a robot arm was moved a total distance of 17 cm. The architectural and system features such as power, mechanical comprise, and redundancy have been improved. It should be noted that sensor design is strongly related with circuit design options, particularly in sensory-interactive control technique.

In conclusion, as complexity increases it is necessary to study the effects of energy in micro-sensor circuits to develop accurate design models. Therefore, the sensor and sensing algorithm has an important role not only in the robot design field, but also in future sensor network applications.

## Figures and Tables

**Table 1.** A notation list of sensor detection and configuration.

**Parameter values**	**2-D Configurable**	**3-D Configurable**
**Sensor node**	c1, c2 : (x1, y1, z1) (x2, y2, z2)	c1, c2, c3, c4 : (x1, y1, z1) (x2, y2, z2) (x3, y3, z3) (x4, y4, z4)
**Target object**	c0 : (x0, y0, z0)	c0 : (x0, y0, z0)
**Scalar of shortest path between sensor node and target object (Distance)**	S1, S2	S1, S2, S3, S4
**Direction of shortest path between sensor node and target object(Pattern)**	*P⃗_a_*, *P⃗_b_*	*P⃗_a_*, *P⃗_b_*

**Table 2.** Power consumption of benchmarking with various values.

**Modes**	**Moving Distance (cm)**	**Total Time (μsec)**	**Power Consumption (mW)**	**PD Product (Pico Joules)**
Moving Mode	7.62	2.10	1.45	3.05
Self Locking Mode	5.33	0.80	1.38	1.10
Level Setting Mode	3.55	0.40	1.25	0.50
Forcing Mode	0.50	0.10	1.21	0.12
